# Anti-MDA5 antibody IgG1 subtype is associated with rapidly progressive interstitial lung disease in anti-MDA5-positive dermatomyositis

**DOI:** 10.1186/s13023-025-03921-y

**Published:** 2025-08-15

**Authors:** Yun Wu, Yue Wang, Yulu Qiu, Chengying Lv, Yujing Zhu, Lei Wang, Lingxiao Xu, Hanxiao You, Fang Wang, Wenfeng Tan

**Affiliations:** 1 Department of Rheumatology, The First Affiliated Hospital of Nanjing Medical University, 300 Guangzhou Road, Nanjing, 210029 Jiangsu China; 2 Department of Hematology and Rheumatology, Huai’an Hospital of Huai’an City, 19 Shanyang Avenue, Huaian, 223200 Jiangsu China; 3 Department of Cardiology, The First Affiliated Hospital of Nanjing Medical University, 300 Guangzhou Road, Nanjing, 210029 Jiangsu China

**Keywords:** Anti-MDA5 antibody, IgG1, Rapidly progressive interstitial lung disease, Indirect immunofluorescence

## Abstract

**Background:**

Rapidly progressive interstitial lung disease (RP-ILD) is a severe, often fatal complication in patients with anti-melanoma differentiation-associated gene 5 antibody-positive dermatomyositis (MDA5^+^ DM). Early prediction of RP-ILD still remains challenging. We aimed to explore the link between anti-MDA5 IgG subtypes and ILD prognosis in individuals with MDA5^+^ DM.

**Methods:**

In a retrospective study involving 71 MDA5^+^ DM-ILD patients, initial serum titers of anti-MDA5 IgG subtypes were measured using indirect immunofluorescence. We then analyzed the associations between these IgG subclasses and the development of RP-ILD.

**Result:**

Of the 71 patients, 30% developed RP-ILD. RP-ILD patients had a shorter disease course and a higher mortality rate than non-RP-ILD patients (both *P* < 0.001). A notable link was found between RP-ILD and anti-MDA5 IgG1 (*P* < 0.05), with 100% of RP-ILD patients showing IgG1 titers ≥ 1:100. Additionally, IgG3 positivity was more prevalent in RP-ILD (*P* < 0.05). Multivariate logistic regression analysis identified high titers of anti-MDA5 IgG1 and a high neutrophil–lymphocyte ratio (NLR^high≥5.22^) as independent risk factors for RP-ILD (*P* = 0.020, 0.017, respectively). The combination of anti-MDA5 IgG1 ≥ 1:100 with an NLR ≥ 5.22 improved the predictive accuracy for RP-ILD, yielding an AUC of 0.80.

**Conclusions:**

Elevated anti-MDA5 IgG1 titers are strongly related to RP-ILD in MDA5^+^ DM and function as an important marker for early detection of individuals at high risk. Combining anti-MDA5 IgG1 levels with NLR further enhances predictive accuracy for RP-ILD, offering a practical approach for clinical monitoring and early intervention.

**Supplementary Information:**

The online version contains supplementary material available at 10.1186/s13023-025-03921-y.

## Introduction

Anti-melanoma differentiation-associated gene 5 antibody positive dermatomyositis (MDA5^+^ DM) is strongly related to interstitial lung disease (ILD) [1–3]. Among MDA5^+^ DM patients, ILD occurs in up to 95% of cases, with about 30% progressing to rapidly progressive ILD (RP-ILD). RP-ILD involves worsening lung changes, increasing breathlessness, severe low oxygen levels, and poor response to immunosuppressants, resulting in a 60% mortality rate within 6 months [4, 5]. Therefore, identifying reliable biomarkers for early RP-ILD prediction is vital for improving survival in MDA5^+^ DM patients. 

Over the past decade, considerable efforts have been directed toward identifying prognostic biomarkers related to MDA5^+^ DM. The anti-Ro52 antibody, frequently linked with myositis, is often found in ILD across autoimmune conditions [6, 7]. In MDA5^+^ DM, its presence is strongly linked to RP-ILD and higher mortality risk [6, 7]. Additionally, Type I interferon (IFN) and its signatures are reported to be highly expressed in the skin, blood, lungs, and muscle tissues of MDA5^+^ DM patients [8–10]. The Type I IFN score, based on four gene expressions (IFI44, Mx1, MxA, and IRF1), has proven valuable in observing disease progression and estimating mortality in these patients [11]. Furthermore, peripheral lymphocyte counts and serum markers like ferritin, KL-6, SPP1, and BAFF are linked to disease activity and unfavorable outcomes in MDA5^+^ DM [12–16].

The anti-MDA5 antibody was discovered in the blood of individuals diagnosed with clinically amyopathic dermatomyositis (CADM) [17]. It was subsequently linked to RP-ILD, and the antigen CADM-140 was later determined to be MDA5 in 2009 [18]. Studies have focused on various aspects of the MDA5 antibody, including its titers, which have been reported to correlate with RP-ILD, mortality, treatment response, and relapse risk [19–21]. Anti-MDA5 antibody levels typically decrease or even normalize following remission [22, 23]. However, some studies suggest that anti-MDA5 antibody titers alone may not reliably predict prognosis or treatment response in MDA5^+^ DM patients [24, 25]. Additionally, not every patient with anti-MDA5 antibodies experiences RP-ILD, indicating the need to explore other risk factors. 

Recent studies have also examined specific patterns of the anti-MDA5 antibody. A small study found that a specific immunofluorescence pattern in Hep-2 cells is linked to a higher risk of RP-ILD in individuals with MDA5^+^ DM [26]. Additionally, two other studies found associations between different IgG subclasses of the anti-MDA5 antibody and mortality in these individuals, as measured by enzyme-linked immunosorbent assay (ELISA), suggesting a link between IgG subtypes and disease severity [27, 28]. However, these studies were limited by small sample sizes and focused primarily on mortality outcomes rather than on the early prediction of RP-ILD.

Given the critical need for early prediction of RP-ILD to inform therapeutic decisions in clinical practice, our study used indirect immunofluorescence (IIF) to analyze anti-MDA5 IgG subtype distribution and titers in MDA5^+^ DM-ILD patients and evaluated their diagnostic significance for RP-ILD. We also investigated the potential of combining anti-MDA5 IgG subtypes with other laboratory markers for predicting RP-ILD. 

## Methods

### Patients

A retrospective analysis evaluated 71 MDA5^+^ DM patients with ILD at the First Affiliated Hospital of Nanjing Medical University between March 2019 and September 2023. All individuals fulfilled the European NeuroMuscular Center or Sontheimer standards for DM [29–31], and tested positive for anti-MDA5 IgG by immunoblotting (EUROIMMUN, Lübeck, Germany). Patients were diagnosed with ILD if they met all the following criteria: (1) respiratory symptoms including dry cough, wheezing and exertional dyspnea; (2) physical signs revealing Velcro crackles in the lung bases, and clubbing; (3) high-resolution CT (HRCT) scans showing ground-glass opacities, consolidations, reticulations and/or honeycombing; (4) lung function tests indicating restrictive impairments (total lung capacity and diffusing capacity of carbon monoxide [DLCO] < 80% of predicted), excluding infection and drug-induced interstitial changes [32]. The definition of RP-ILD was based on previously reported criteria, with some modification. Briefly, RP-ILD was defined as experiencing any of the following four conditions within one month after DM symptom onset: (1) acute and progressive worsening of dyspnea requiring admission to the hospital or extra oxygen support; (2) progressive impairment of lung function, including over 10% drop in forced vital capacity (FVC) or more than 15% decrease in DLCO with reduced FVC; (3) radiological progression of HRCT (over 20% increase in interstitial abnormalities of the lung); (4) arterial blood gas abnormalities (results indicating respiratory failure or a drop in oxygen partial pressure exceeding 10 mmHg) [17, 33].

Serum samples were collected at each patient's initial hospital admission and stored at − 80 °C. Survival status and clinical data were sourced from electronic records and confirmed via phone or hospital records, with the study concluding on March 11, 2024. All patients signed a written informed consent form before enrolment in this study. The study received approval from the Research and Ethics Committees of the First Affiliated Hospital of Nanjing Medical University (ID: 2020-SR-265).

### Determination of anti-MDA5 IgG subclasses

IgG subclass antibodies in serum samples were assessed using the Anti-MDA5 IgG Subtype Antibodies Profile Detection test (IIF) (Beijing HumaDX Tech Co., Ltd., Beijing, China). The serum was diluted 1:10 with a sample diluent and mixed thoroughly. Then, one hundred microliters (100 µl) of 1 × phosphate buffer saline (PBS) were added to each of the four reaction wells on the antigen slide, left for 5 min, and removed. Next, 100 µl of the diluted serum was added to each well, incubated at room temperature for 30 min in a humid chamber for antibody binding, then washed with 1 × PBS and soaked for 5 min to remove nonspecifically bound antibodies. Excess liquid was removed from the reaction wells, and 50 µl of Alexa Fluor 488-conjugated anti-human IgG1, IgG2, IgG3, and IgG4 secondary antibodies were added. The mixture was then incubated in a dark, humid chamber at room temperature for 30 min to ensure specific binding to the primary antibodies. The slides were washed again in the same manner as before. Finally, the results were promptly observed under a fluorescence microscope and interpreted by two qualified professionals.

### Statistical analysis

Counts and percentages represented categorical variables, while continuous variables were displayed as means (SD) or medians (IQR). Categorical comparisons used the chi-square or Fisher's exact test. Skewness and kurtosis z-scores assessed continuous variable distribution. The Mann–Whitney U test was used for non-normal distributions, and the unpaired Student's t-test for normal distributions in two-group analyses. Survival probabilities were assessed using the Kaplan–Meier method, and group comparisons were made with the wilcoxon test. Binary logistic regression identified risk factors. The receiver operating characteristic curve (ROC) evaluated RP-ILD prediction accuracy, with area under the curve (AUC) determining the best diagnosis. Analyses were conducted with SPSS 26.0, and graphs were created using Prism 8.0. *P* < 0.05 was statistically significant.

## Results

### Baseline features of individuals with MDA5^+^ DM-ILD

Table 1 presents the baseline clinical and lab data for 71 MDA5^+^ DM-ILD patients. Of these, 21 (30%) developed RP-ILD during 60 months of follow-up. RP-ILD patients experienced a shorter period of disease (2.5 months vs 20.0 months, *P* < 0.001) and an elevated rate of mortality (86% vs 16%,* P* < 0.001) than those with non-RP-ILD. Moreover, the concentrations of C-reactive protein (CRP) [median (IQR) 9.4 (2.9–23.5) vs 4.5 (2.4–7.5) mg/l, *P* = 0.032] and ferritin [median (IQR) 968.5 (853.4–1231.4) vs 538.6 (215.3–906.9) ng/ml, *P* = 0.011] were notably elevated in the RP-ILD patients, compared to individuals without RP-ILD. Lymphocyte counts did not show significant variation between the two groups. However, neutrophil counts were ralatively increased in RP-ILD patients [median (IQR) 4.86 (3.46–7.28) vs 3.45 (2.56–4.63) × 10^9^/L, *P* = 0.036]. Correspondingly, a significantly increased neutrophil–lymphocyte ratio (NLR) was observed in individuals with RP-ILD than those without it [median (IQR) 8.14 (4.22–11.85) vs 4.15 (3.22–5.94), *P* = 0.015].

**Table 1:** Clinical characteristics of MDA5^+^ DM patients with ILD

Characteristic	MDA5^+^ DM−ILD		*t*/*z*/*x*^2^/Fisher	*P*-value
	Non-RP-ILD (*n* = 50)	RP-ILD (*n* = 21)		
*General information*				
Sex: female, *n* (%)	36 (72)	13 (62)	0.705	0.401
Age, mean (S.D.), years	50 (1.7)	55 (2.6)	− 1.725	0.089
Deceased, *n* (%)	8 (16)	18 (86)	30.967	< 0.001**
Disease duration, median (IQR), months	20 (7.8–33)	2.5 (1–4)	− 5.021	< 0.001**
*Clinical manifestations*				
Muscle weakness, *n* (%)	18 (36)	8 (38)	0.028	0.867
Skin ulcers, *n* (%)	6 (12)	3 (14)	–	1.000
Heliotrope rash, *n* (%)	27 (54)	10 (48)	0.241	0.623
Gottron’s sign, *n* (%)	33 (66)	10 (48)	2.092	0.148
V-sign, *n* (%)	19 (38)	9 (43)	0.146	0.702
Shawl sign, *n* (%)	11 (22)	5 (24)	–	1.000
Erythema of the skin, *n* (%)	16 (32)	5 (24)	0.476	0.490
Mechanic’s hands, *n* (%)	20 (40)	7 (33)	0.279	0.597
Periungual erythema, *n* (%)	12 (24)	3 (14)	–	0.527
Arthritis, *n* (%)	11 (22)	6 (29)	0.351	0.554
Raynaud's phenomenon, *n* (%)	0 (0)	1 (5)	–	0.296
Translocation calcification, *n* (%)	1 (2)	0 (0)	–	1.000
*Laboratory features*				
*Anti-MDA5 IgG antibody, IB, n (%)*			− 0.721	0.471
Positive (+)	12 (24)	6 (29)		
Moderate positive (++)	9 (18)	5 (24)		
Strong positive (+++)	29 (58)	10 (48)		
*Anti-Ro52 IgG antibody, IB, n (%)*			− 1.128	0.259
Negative (−)	13 (26)	2 (10)		
Positive (+)	14 (28)	7 (33)		
Moderate positive (++)	7 (14)	4 (19)		
Strong positive, (+++)	16 (32)	8 (38)		
WBC, median (IQR), 10^9^/l	4.89 (4.06–6.34)	6.67 (4.57–8.39)	− 1.844	0.065
L, median (IQR), 10^9^/l	0.85 (0.55–1.11)	0.78 (0.45–1.04)	− 1.102	0.270
M, mean (S.D.), 10^9^/l	0.49 (0.22)	0.47 (0.27)	0.31	0.758
N, median (IQR), 10^9^/l	3.45 (2.56–4.63)	4.86 (3.46–7.28)	− 2.094	0.036*
NLR, median (IQR)	4.15 (3.22–5.94)	8.14 (4.22–11.85)	− 2.432	0.015*
PLT, median (IQR), 10^9^/l	182 (156–234.5)	177 (156–247)	− 0.162	0.872
ALT, median (IQR), U/l	55.4 (28.8–94.2)	39.95 (21.38–76.93)	− 0.957	0.339
AST, median (IQR), U/l	52.2 (29.9–83.6)	56.5 (29.8–80.8)	− 0.111	0.912
FIB, median (S.D.), g/l	3.25 (0.87)	3.57 (0.86)	− 1.346	0.184
D-Dimer, median (IQR), mg/l	0.81 (0.48–1.34)	0.83 (0.48–1.89)	− 0.659	0.510
IgG, median (IQR), g/l	13.3 (11.1–15.9)	12.6 (9.9–15.5)	− 0.878	0.380
IgA, mean (S.D.), g/l	2.82 (1.30)	2.80 (0.80)	0.049	0.961
IgM, median (IQR), g/l	1.21 (0.84–1.72)	1.18 (0.91–1.47)	− 0.025	0.980
LDH, median (IQR), IU/l	286 (239–364)	344 (256–409)	− 1.390	0.165
CK, median (IQR), IU/l	42 (31–114)	70 (27–116)	− 0.395	0.693
C3, mean (S.D.), g/l	0.83 (0.18)	0.90 (0.24)	− 1.199	0.235
C4, mean (S.D.), g/l	0.25 (0.07)	0.26 (0.07)	− 0.717	0.476
CRP, median (IQR), mg/l	4.5 (2.4–7.5)	9.4 (2.9–23.5)	− 2.145	0.032*
ESR, mean (S.D.), mm/h	34 (18)	42 (23)	− 1.294	0.201
Ferritin, median (IQR), ng/ml	538.6 (215.3–906.9)	968.5 (853.4–1231.4)	− 2.546	0.011*

*P*-values were determined using the unpaired *t*-test, Mann-Whitney U test, *χ*^2^ test or Fisher’s exact test

IB, immunoblotting; WBC, white blood cell; L, lymphocyte; M: monocyte; N, neutrocyte; NLR, neutrophil–lymphocyte ratio; PLT, platelet; ALT, alanine aminotransferase; AST, aspartate aminotransferase; FIB, fibrinogen; LDH, lactic dehydrogenase; CK, creatine kinase; CRP, C-reactive protein; ESR, erythrocyte sedimentation rate; DM-ILD, dermatomyositis with interstitial lung disease; Non-RP-ILD, Non-rapidly progressive interstitial lung disease; RP-ILD, rapidly progressive interstitial lung disease

*P < 0.05

**P < 0.01

### Distribution of anti-MDA5 IgG subtypes in MDA5^+^ DM-ILD

**Fig. 1:**
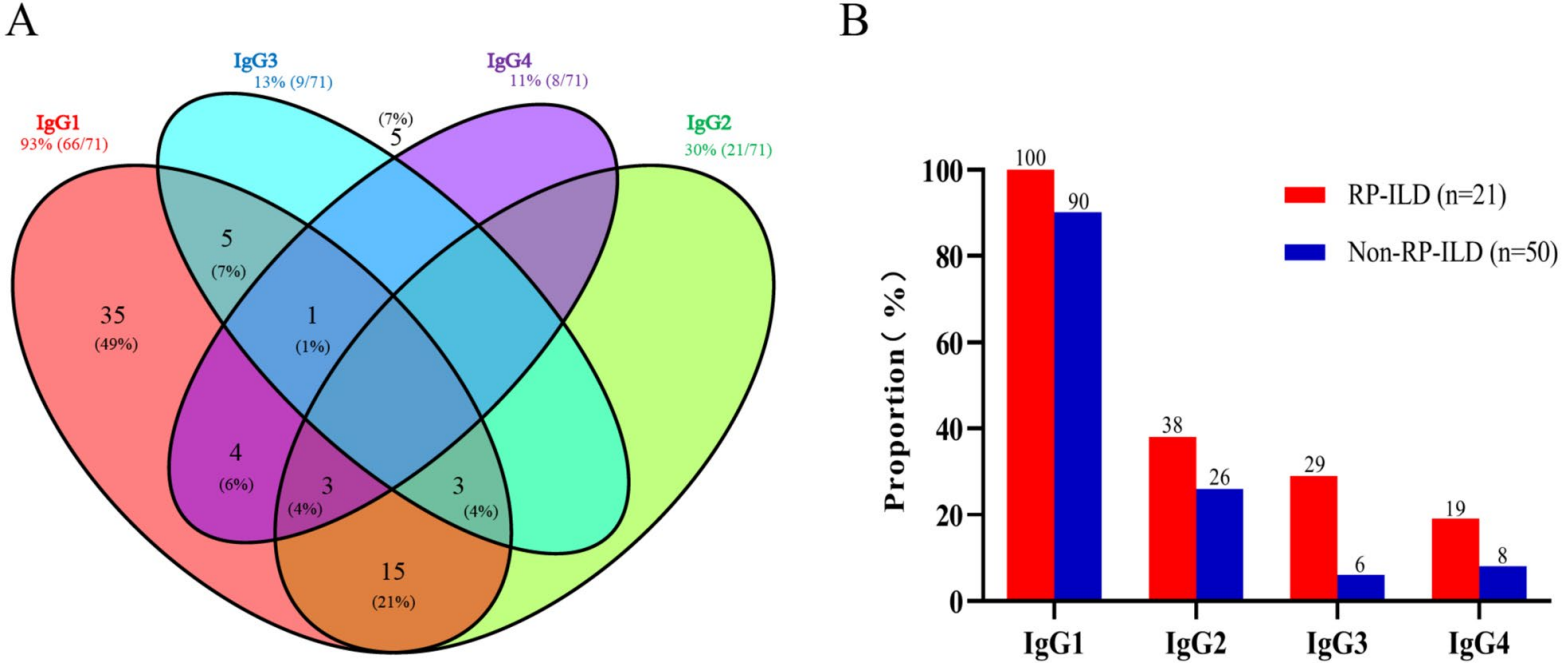
Distribution of anti-MDA5 IgG subclasses in MDA5^+^ DM-ILD patients. **A** Proportions of anti-MDA5 IgG subclasses (IgG1, IgG2, IgG3, and IgG4) at an antibody titer threshold of 1:10 across 71 MDA5^+^ DM-ILD patients. **B** Comparison of the prevalence of anti-MDA5 IgG subclasses between 21 patients with rapidly progressive ILD (RP-ILD) and 50 patients with non-RP-ILD. A titer of 1:10 or greater is considered positive for anti-MDA5 IgG subclasses

Since anti-MDA5 IgG was positive in all individuals, we then examined its subclasses. The distribution of IgG subtypes specific to anti-MDA5 is depicted in Fig. 1A, with titers of ≥ 1:10 considered positive. Among the 71 individuals with MDA5^+^ DM-ILD, IgG1 appeared most commonly, found in 93% of cases, followed by IgG2 (30%), IgG3 (13%), and IgG4 (11%). IgG1 positivity can occur alone in 49% of cases, while 34% of patients may exhibit double positivity and 10% may show triple positivity. Approximately 7% of patients were negative for any anti-MDA5 IgG subclass (Fig. 1A). When individuals with MDA5^+^ DM-ILD were categorized into non-RP-ILD and RP-ILD subgroups, the prevalence of anti-MDA5 IgG1, IgG2, IgG3, and IgG4 was 100, 38, 29, and 19% in the RP-ILD subgroup, compared to 90, 26, 6, and 8% in the control group (Fig. 1B).

### Association of anti-MDA5 IgG subclass titers with RP-ILD in MDA5^+^ DM-ILD

The above results indicate that when an anti-MDA5 IgG subclass titer of 1:10 is defined as positive, IgG1 appears to be more prevalent among patients with RP-ILD. We then investigated whether the different titers of anti-MDA5 IgG subtypes are linked to disease severity (Table 2). A clear titer-dependent relationship between IgG1 and RP-ILD was observed, differing from those without RP-ILD (*P* = 0.007). Among RP-ILD patients, 29% had an IgG1 titer of 1:320, and 62% had a titer of ≥ 1:1000, which is significantly higher than the 10 and 40% observed in the individuals with non-RP-ILD, respectively. Notably, none of the MDA5^+^ DM-ILD individuals who developed RP-ILD had IgG1 titers lower than 1:100. Using a 1:100 cut-off for antibody titer, 100% of RP-ILD patients had IgG1 titers ≥ 1:100, compared to only 64% in those without RP-ILD (*P* = 0.001).

**Table 2:** Comparison of anti-MDA5 IgG subtype titers between Non-RP-ILD and RP-ILD

Anti-MDA5 IgG subtypes	MDA5^+^ DM-ILD		*P*-value
	Non-RP-ILD (*n* = 50)	RP-ILD (*n* = 21)	
*IgG1*			0.007**
Negative (−)	5 (10)	0 (0)	
1:10	6 (12)	0 (0)	
1:32	7 (14)	0 (0)	
1:100	7 (14)	2 (9)	
1:320	5 (10)	6 (29)	
≥ 1:1000	20 (40)	13 (62)	
*IgG2*			0.388
Negative (−)	37 (74)	13 (62)	
1:10	0 (0)	2 (10)	
1:32	9 (18)	3 (14)	
1:100	2 (4)	3 (14)	
1:320	2 (4)	0 (0)	
≥ 1:1000	0 (0)	0 (0)	
*IgG3*			0.008**
Negative (−)	47 (94)	15 (71)	
1:10	1 (2)	1 (5)	
1:32	1 (2)	1 (5)	
1:100	1 (2)	4 (19)	
1:320	0 (0)	0 (0)	
≥ 1:1000	0 (0)	0 (0)	
*IgG4*			0.191
Negative (−)	46 (92)	17 (81)	
1:10	1 (2)	0 (0)	
1:32	0 (0)	2 (9.5)	
1:100	3 (6)	2 (9.5)	
1:320	0 (0)	0 (0)	
≥ 1:1000	0 (0)	0 (0)	
*IgG1*			
≥ 1:100	32 (64)	21 (100)	0.001**
*IgG2*			
≥ 1:100	4 (8)	3 (14)	0.415
*IgG3*			
≥ 1:100	1 (2)	4 (19)	0.025*
*IgG4*			
≥ 1:100	3 (6)	2 (9.5)	0.629

*P*-values were determined using the Mann–Whitney U-test, *χ*^2^ or Fisher’s exact test

DM-ILD, dermatomyositis with interstitial lung disease; Non-RP-ILD, Non-rapidly progressive interstitial lung disease; RP-ILD, rapidly progressive interstitial lung disease

*P < 0.05

**P < 0.01

Similarly, individuals with RP-ILD were more prone to having IgG3 (titers ≥ 1:100) than those with non-RP-ILD (19% vs 2%, *P* = 0.025). In comparison, IgG2 and IgG4 titers did not exhibit a significant relationship with RP-ILD (both *P* > 0.05, as shown in Table 2). These results indicate that high titers of IgG1 and IgG3 are strongly correlated with the presence of RP-ILD.

**Fig. 2:**
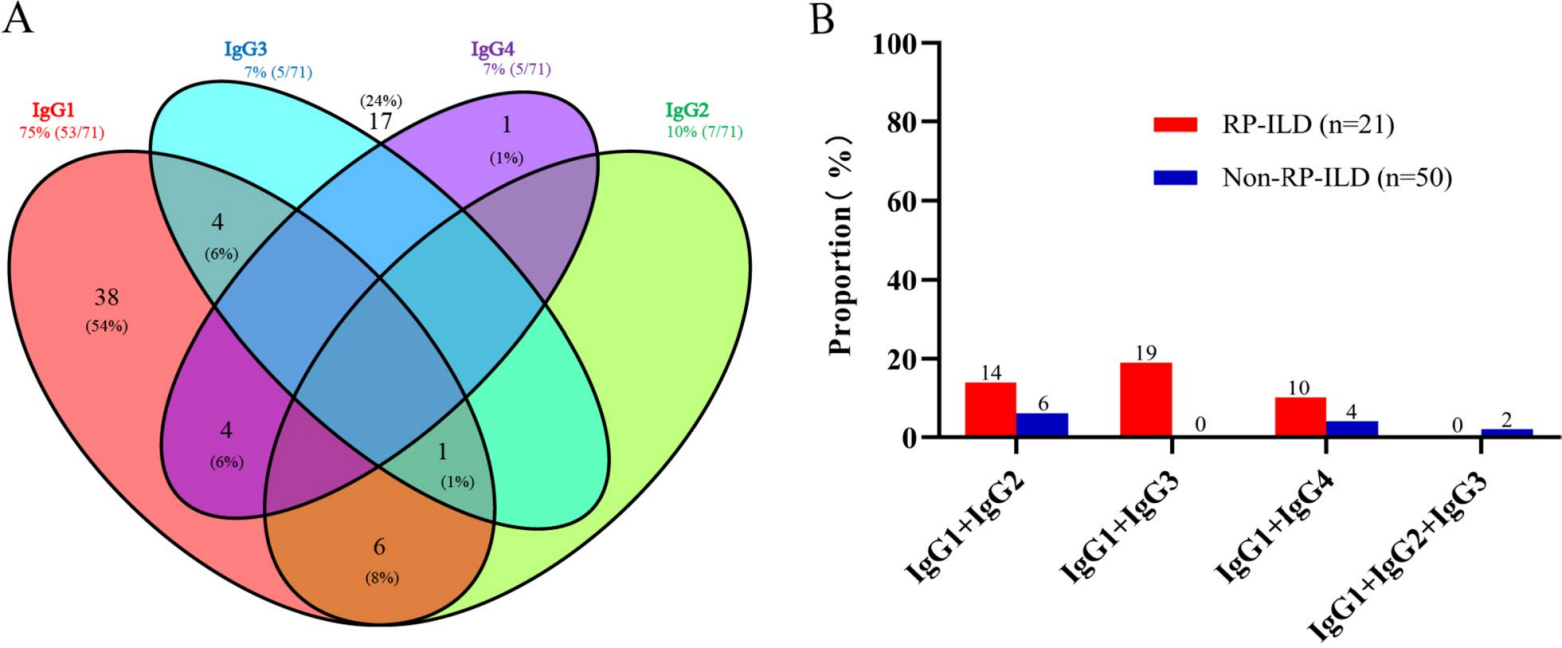
Profiles of anti-MDA5 IgG subclass combinations in RP-ILD and non-RP-ILD patients. **A** Distribution of anti-MDA5 IgG1, IgG2, IgG3, and IgG4 titers at ≥ 1:100 across the 71 MDA5^+^ DM-ILD patients. **B** Different combinations of anti-MDA5 IgG subclasses present in RP-ILD versus non-RP-ILD patients. Bar chart displays patient numbers for each subclass combination group

Furthermore, the titers of anti-MDA5 IgG1 and IgG3 ≥ 1:100 were found in 75% and 7% of sera, respectively, whereas IgG2 and IgG4 were found in 10 and 7% of sera (Fig. 2A). The combination of 2 or 3 subclasses of anti-MDA5 IgG (titers ≥ 1:100) was observed in 14 (20%) patients and 1 (1%) patient, respectively (Fig. 2A). When MDA5^+^ DM-ILD patients were sorted into RP-ILD and non-RP-ILD subgroups, the combinations of anti-MDA5 IgG1 IgG2, IgG1 IgG3, IgG1 IgG4, and IgG1 IgG2 IgG3 were 14, 19, 10, and 0% in the RP-ILD group, compared to 6, 0, 4, and 2% in the non-RP-ILD group (Fig. 2B).

### Anti-MDA5 IgG1 as an independent predictor for RP-ILD

The binary logistic regression model was then employed to assess the association between anti-MDA5 IgG1, IgG3, and other clinically significant parameters identified in Table 1 with RP-ILD (Table 3). Initial univariate logistic regression analysis indicated that both IgG1 [odds ratio (OR) 2.031 (95% CI: 1.215, 3.394), *P* = 0.007] and IgG3 [OR 2.232 (95% CI: 1.156, 4.308), *P* = 0.017] were associated to an elevated risk of RP-ILD. In the subsequent multivariate logistic regression model, we included variables with *P* < 0.1 from the univariate analysis, key antibody (anti-MDA5 IgG, IB), and potential confounders (anti-Ro52 IgG, age, gender). We found that high titers of anti-MDA5 IgG1 [OR 2.296 (95% CI: 1.138, 4.630), *P* = 0.020] independently raise the likelihood of developing RP-ILD. However, IgG3 and anti-MDA5 IgG (IB test) were not independently associated with RP-ILD. In addition, NLR ≥ 5.22 [OR 7.759 (95% CI: 1.434, 41.989), *P* = 0.017, Table 3 and Additional file 1: Fig. S1] and CRP > 8 mg/l [OR 7.321 (95% CI: 1.442, 37.171), *P* = 0.016] were also standalone risk factors for RP-ILD.

**Table 3:** Laboratory indicators associated with RP-ILD in logistic regression model

Variable	Univariable logistic regression			Multivariable logistic regression		
	*β*	OR (95%CI)	*P*-value	*β*	OR (95%CI)	*P*-value
Anti-MDA5 IgG1, IIF	0.709	2.031 (1.215, 3.394)	0.007**	0.831	2.296 (1.138, 4.630)	0.020*
Anti-MDA5 IgG3, IIF	0.803	2.232 (1.156, 4.308)	0.017*	0.244	1.277 (0.525, 3.106)	0.590
Anti-MDA5 IgG, IB	− 0.206	0.814 (0.449, 1.475)	0.498	0.118	1.125 (0.404, 3.130)	0.822
Anti-Ro52 IgG, IB	0.257	1.293 (0.823, 2.029)	0.265	0.214	1.239 (0.625, 2.455)	0.540
Age, ≥50 years	0.613	1.846 (0.637, 5.347)	0.259	0.186	1.204 (0.271, 5.359)	0.807
Gender, male	0.459	1.582 (0.540, 4.639)	0.403	0.578	1.782 (0.331, 9.605)	0.501
NLR^high≥5.22^	1.568	4.798 (1.512, 15.228)	0.008**	2.049	7.759 (1.434, 41.989)	0.017*
CRP, > 8 mg/l	1.512	4.537 (1.432, 14.377)	0.01*	1.991	7.321 (1.442, 37.171)	0.016*
SF, > 300 ng/ml	1.338	3.810 (0.766, 18.959)	0.102			
LDH, > 300 IU/l	0.868	2.381 (0.783, 7.237)	0.126			

Binary logistical regression analyses were used in regression equation. The upper limit of the normal range for continuous variables (CRP, SF, LDH) in healthy individuals served as the cut-off value for binary classification

RP-ILD, rapidly progressive interstitial lung disease; OR, odds ratio; IIF, indirect immunofluorescence; IB, immunoblotting; NLR^high≥5.22^, neutrophil–lymphocyte ratio (high ≥ 5.22); CRP, C-reactive protein; SF, serum ferritin; LDH, lactate dehydrogenase

*P < 0.05

**P < 0.01

Ultimately, to understand the features of patients with anti-MDA5 IgG1, we analyzed clinical and lab parameters between groups with anti-MDA5 IgG1 levels of ≥ 1:100 and < 1:100 (Additional file 2: Table S1). As expected, the frequency of RP-ILD (*P* = 0.001) and mortality (*P* = 0.042) were significantly greater in the ≥ 1:100 patients than in the < 1:100 individuals. A significantly extended median disease duration was seen in the < 1:100 individuals (21.5 months) compared to the ≥ 1:100 patients (8 months) (*P* = 0.021). Skin erythema occurred more often in the individuals with a ratio of < 1:100 (50% vs 23%, *P* = 0.028). No substantial changes were detected in the other clinical and lab indicators.

### Predictive value of anti-MDA5 IgG1 in combination with key laboratory indicators in RP-ILD

**Fig. 3:**
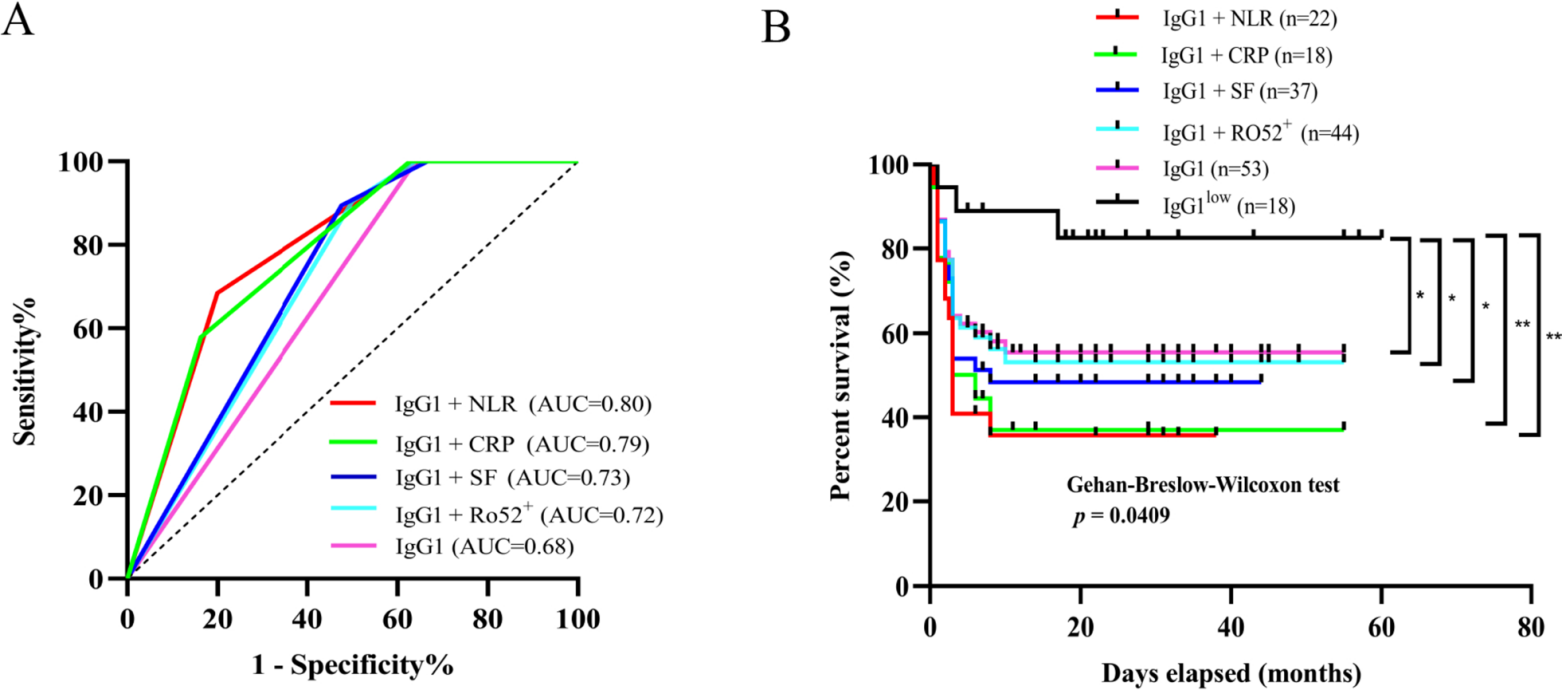
Diagnostic value of anti-MDA5 IgG1 in combination with key laboratory indicators in predicting RP-ILD. **A** Receiver Operating Characteristic (ROC) curves showing the Area Under the Curve (AUC) for the predictive power of anti-MDA5 IgG1 combined with the neutrophil–lymphocyte ratio (NLR), C-reactive protein (CRP), serum ferritin (SF), anti-Ro52 antibody (Ro52), or anti-MDA5 IgG1 alone (≥ 1:100) for RP-ILD development. **B** Survival analysis using the wilcoxon test to compare RP-ILD outcomes in patients with high anti-MDA5 IgG1 in combination with NLR, CRP, SF, Ro52, or high anti-MDA5 IgG1 alone (≥ 1:100) versus low anti-MDA5 IgG1 (< 1:100). ^*^P < 0.05, ^**^P < 0.01

Aiming to further enhance the predictive accuracy for RP-ILD, we used the ROC curves to evaluate the discriminative capacities of high titers of anti-MDA5 IgG1 (≥ 1:100) in combination with an elevated of NLR, CRP, anti-Ro52 antibody, and serum ferritin (SF). Anti-MDA5 IgG1 alone could predict RP-ILD [AUC 0.68 (95% CI: 0.56, 0.80), Fig. 3A]. Anti-MDA5 IgG1 coupled with NLR^high≥5.22^ showed the highest AUC in the ROC analysis to predict RP-ILD [AUC 0.80 (95% CI: 0.69, 0.91), Fig. 3A]. Furthermore, combining anti-MDA5 IgG1 with CRP^high >8 mg/l^ [AUC 0.79 (95% CI: 0.67, 0.90)], anti-Ro52 antibody [AUC 0.72 (95% CI: 0.60, 0.84)] and SF^high >300 ng/ml^ [AUC 0.73 (95% CI: 0.60, 0.85)] can also enhance the predictive accuracy for RP-ILD (Fig. 3A). Using the Kaplan–Meier survival analysis, the MDA5^+^ DM-ILD individuals with anti-MDA5 IgG1 ≥ 1:100 and NLR ≥ 5.22 at baseline had significantly the lowest survival (wilcoxon test, *P* = 0.0409, Fig. 3B).

## Discussion

RP-ILD is a fatal complication in MDA5^+^ DM, making early detection crucial for better prognosis and informed treatment decisions [4, 5]. This study sheds light on the link between anti-MDA5 IgG subtypes and ILD prognosis in MDA5^+^ DM. Our findings indicate that anti-MDA5 IgG1 independently contributes to the risk of RP-ILD, underscoring the clinical importance of anti-MDA5 IgG subclasses in identifying high risk patients at an early stage.

While previous studies have explored the link between various anti-MDA5 antibody subclasses and disease outcomes [27, 28]. Our research offers three significant contributions that enhance the existing literature. First, We are the pioneers in identifying a direct link between anti-MDA5 IgG1 and RP-ILD in individuals with MDA5^+^ DM. Given that RP-ILD is a major reason for death among these patients, early identification of individuals at high risk could optimize diagnosis and treatment timing, potentially preventing disease progression and reducing mortality rates. Second, our study demonstrates that combining anti-MDA5 IgG1 with simple clinical parameters, such as the NLR, significantly enhances both the sensitivity and specificity of RP-ILD prediction. This integrated approach could facilitate more accurate early detection in clinical settings. Lastly, while prior studies relied on ELISA to detect anti-MDA5 antibodies, we employed indirect immunofluorescence, a method that provides a more sensitive and specific means of identifying different anti-MDA5 IgG subclasses.

Earlier studies indicated that anti-MDA5 IgG1^+^IgG4^+^ or IgG1^+^IgG3^+^ antibodies were linked to poor outcomes in MDA5^+^ DM individuals [27, 28]. In our study, we similarly found higher rates of positivity for anti-MDA5 IgG1 and IgG3 in RP-ILD. The positivity rate (titers ≥ 1:100) of anti-MDA5 IgG1 was 75% in the 71 patients, consistent with the reported positivity rate of 72% [27]. Additionally, IgG4 showed no significant difference between RP-ILD patients and non-RP-ILD patients. In the multivariate regression analysis, IgG3 was not found to be an independent risk factor for RP-ILD, whereas IgG1 was. Given that MDA5^+^ DM is a rare condition, both our study and other studies have utilized relatively small sample sizes. However, across three distinct studies, a consistent association has been established between the anti-MDA5 IgG1 and disease prognosis. This underscores the vital role of anti-MDA5 IgG1 in the disease process, particularly its association with the development of RP-ILD. The identification of anti-MDA5 IgG1 as an independent predictor for RP-ILD highlights its potential role as a valuable biomarker for early prognostic assessment.

IgG antibodies are vital to the immune system and include four subclasses: IgG1, IgG2, IgG3, and IgG4 [34]. These subclasses are produced in response to distinct antigens. For example, T-dependent protein antigens primarily induce IgG1 and IgG3, while T-independent carbohydrate antigens mostly trigger IgG2. Prolonged antigen exposure, such as in allergic desensitization, leads predominantly to IgG4 production. Among these subclasses, IgG1 is the most prevalent and possesses a highly flexible hinge domain, which provides a strong binding affinity to FcγR. This structural property enhances its capacity for complement activation and antibody-dependent cellular cytotoxicity, making it essential in immune responses [35, 36].

How IgG1 relates to RP-ILD in MDA5^+^ DM is still unknown. Viral infections are often considered triggers for MDA5^+^ DM, with excessive CD8^+^ T-cell activation and the interferon pathway being key molecular mechanisms behind RP-ILD development [9]. It is possible that T-dependent antigens or viral infections may contribute to autoimmune responses and result in the production of IgG1 antibodies [37]. IgG1 can bind more effectively to complement and Fc receptors, thus playing an important role in antibody-dependent cellular cytotoxicity, potentially causing cytokine overproduction and inflammation in the lungs.

Elevated IgG1 levels have been observed in autoimmune diseases [38, 39]. The IgG1 and IgG4 subclasses of antinuclear antibodies (ANA) are chiefly related to skin and kidney involvement in systemic lupus erythematosus (SLE) [40, 41]. Moreover, viral infections commonly trigger both IgG1 and IgG3 production [42]. In people with HIV, higher IgG1 levels are linked to continuous viral replication and immune activation [43]. Elevated IgG1 levels have also been observed in COVID-19, serving as a potential indicator of disease severity [44, 45]. The similarity between MDA5^+^ DM and COVID-19, particularly regarding their high inflammatory states and pulmonary involvement [46], supports our finding that IgG1 was linked to RP-ILD in MDA5^+^ DM.

Therefore, the clinical implications of anti-MDA5 IgG1 subclasses may exceed the scope of prognostic evaluation. They may be linked to the pathophysiological mechanisms of RP-ILD. It is possible that IgG1 plays crucial roles in cell-mediated immune responses and may influence the inflammatory response and lung injury in patients. Further investigations need to target the immunological roles of these subclasses and their contribution to DM-ILD pathogenesis.

NLR serves as a new inflammatory marker, and elevated NLR values are frequently observed in severe viral infections and MDA5^+^ DM-ILD [47, 48]. NLR is more accessible compared to traditional inflammatory markers such as LDH and SF. Our findings demonstrate that combination of anti-MDA5 IgG1 and NLR increases the predictive accuracy for RP-ILD in MDA5^+^ DM-ILD individuals, highlighting the high inflammatory state linked to RP-ILD as we previously reported [19].

Some limitations are present in our study. First, the limited sample size in this research may affect the robustness of the results. Second, the relationship between the anti-MDA5 IgG1 and prognosis was not analyzed in conjunction with treatment responses. Third, we did not measure the total anti-MDA5 IgG titers by IIF and also not perform a dynamic analysis of these IgG subtype titers at different stages of disease progression. Therefore, future studies should involve larger, multi-center, prospective research to further elucidate the connection between anti-MDA5 IgG1 and RP-ILD development.

## Conclusions

Our research confirms a significant link between anti-MDA5 IgG1 and RP-ILD. An anti-MDA5 IgG1 level of ≥ 1:100 independently predicts the risk of developing RP-ILD. Moreover, combining anti-MDA5 IgG1 with NLR provides a straightforward and practical method for improving the diagnosis of RP-ILD. We advise clinicians to closely monitor patients with DM-ILD who exhibit increased levels of anti-MDA5 IgG1 and NLR to quickly identify and manage those at high risk for RP-ILD. Finally, our findings offer fresh perspectives on the clinical diagnostic significance of classifying myositis-specific autoantibodies. 

## Data availability statement

The data supporting the findings of this study are available within the article and its supplementary materials.

## Supplementary Information

Additional file 1.

Additional file 2.
