# Supplementary material for: Anti-MDA5 antibody IgG1 subtype is associated with rapidly progressive interstitial lung disease in anti-MDA5-positive dermatomyositis
Source: Orphanet J Rare Dis. 2025 Aug 15;20:434. doi: 10.1186/s13023-025-03921-y (PMC12357345; doi:10.1186/s13023-025-03921-y)
Supplement: Supplementary file 1 — Additional file 1. [file 13023_2025_3921_MOESM1_ESM.docx]

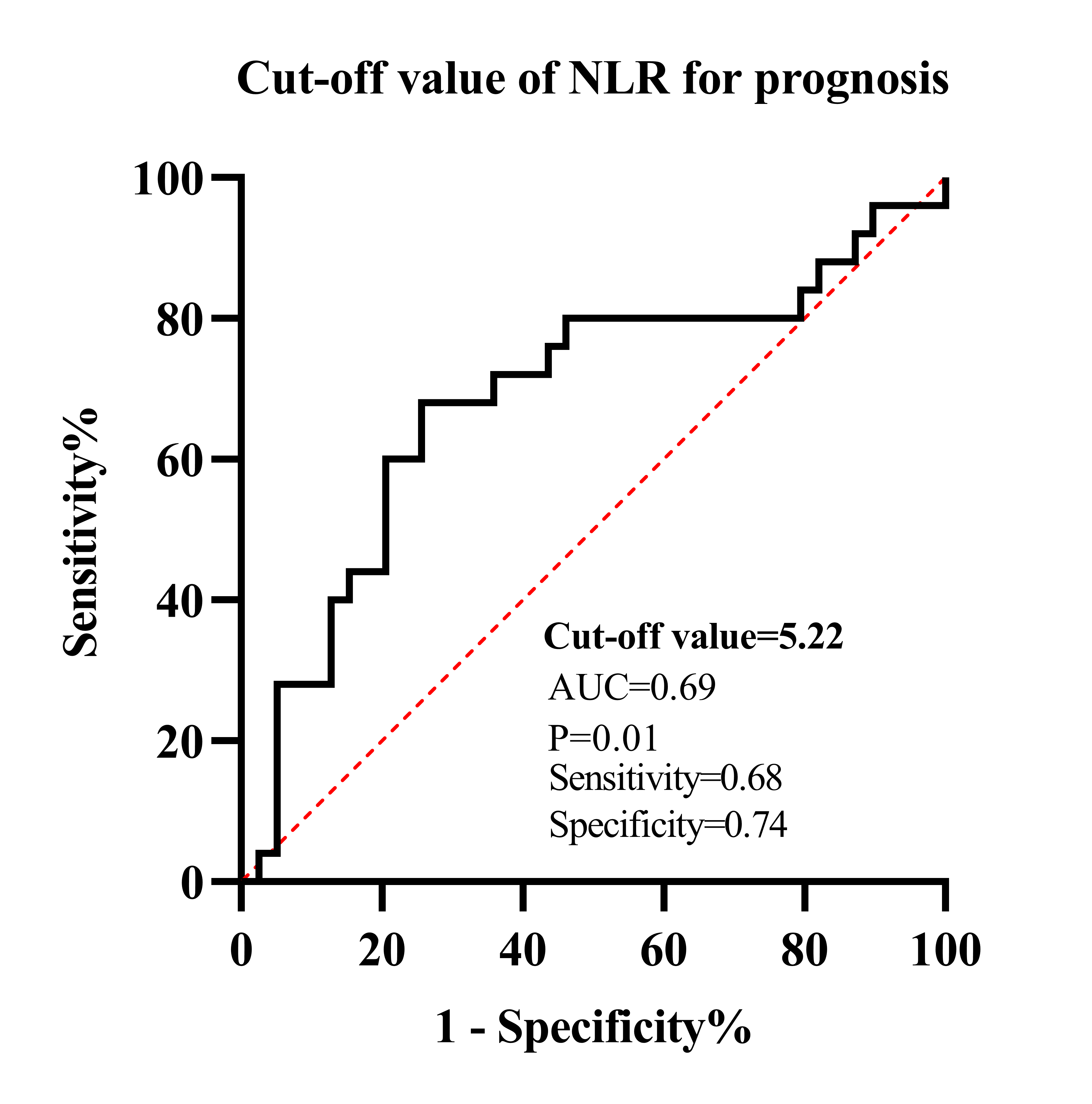


**Fig. S1** ROC curves of NLR for the outcome of death in MDA5^+^ DM-ILD individuals. The receiver operating characteristic (ROC) curve was used to determine the cut-off value of NLR for the death of patients with MDA5^+^ DM-ILD. NLR: neutrophil-lymphocyte ratio; MDA5^+^ DM-ILD: anti-MDA5 positive dermatomyositis with interstitial lung disease.
