# Supplementary material for: Anti-MDA5 antibody IgG1 subtype is associated with rapidly progressive interstitial lung disease in anti-MDA5-positive dermatomyositis
Source: Orphanet J Rare Dis. 2025 Aug 15;20:434. doi: 10.1186/s13023-025-03921-y (PMC12357345; doi:10.1186/s13023-025-03921-y)
Supplement: Supplementary file 2 — Additional file 2. [file 13023_2025_3921_MOESM2_ESM.docx]

**Table S1** Characteristics of clinical and lab indicators in MDA5^+^ DM-ILD individuals with anti-MDA5 IgG1 ≥1:100

| **Characteristic** | **Anti-MDA5 IgG1** | | **t/z/x^2^/Fisher** | **P-value** |
| --- | --- | --- | --- | --- |
|  | **< 1:100 (n=18)** | **≥ 1:100 (n=53)** |  |  |
| Sex:female, n (%) | 13 (72) | 36 (68) | 0.116 | 0.733 |
| Age, mean (S.D.), years | 53 (11) | 51 (13) | 0.611 | 0.543 |
| Deceased, n (%) | 3 (17) | 23 (43) | 4.136 | **0.042^*^** |
| Disease duration, median (IQR), months | 21.5 (14.5-35.5) | 8 (3-29) | -2.316 | **0.021^*^** |
| Muscle weakness, n (%) | 6 (33) | 20 (38) | 0.112 | 0.738 |
| Skin ulcers, n (%) | 2 (11) | 7 (13) | - | 1.000 |
| Heliotrope rash, n (%) | 8 (44) | 29 (55) | 0.568 | 0.451 |
| Gottron’s sign, n (%) | 9 (50) | 34 (64) | 1.127 | 0.289 |
| V-sign, n (%) | 5 (28) | 23 (43) | 1.372 | 0.241 |
| Shawl sign, n (%) | 3 (22) | 13 (24) | - | 0.745 |
| Erythema of the skin, n (%) | 9 (50) | 12 (23) | 4.828 | **0.028^*^** |
| Mechanic’s hands, n (%) | 7 (39) | 20 (38) | 0.008 | 0.931 |
| Periungual erythema, n (%) | 4 (22) | 11 (21) | - | 1.000 |
| Arthritis, n (%) | 3 (17) | 14 (26) | - | 0.531 |
| Raynaud's phenomenon, n (%) | 0 (0) | 1 (2) | - | 1.000 |
| Translocation calcification, n (%) | 0 (0) | 1 (2) | - | 1.000 |
| RP-ILD | 0 (0) | 21 (39.6) | 10.128 | **0.001^**^** |
| WBC, median (IQR), 10^9^/l | 4.86 (4.27-6.50) | 5.13 (4.01-7.33) | -0.705 | 0.481 |
| L, median (IQR), 10^9^/l | 0.92 (0.64-1.00) | 0.79 (0.47-1.11) | -0.535 | 0.593 |
| M, median (IQR), 10^9^/l | 0.52 (0.33-0.69) | 0.42 (0.28-0.59) | -0.667 | 0.505 |
| N, median (IQR), 10^9^/l | 3.34 (2.99-4.90) | 3.80 (2.70-5.31) | -0.636 | 0.525 |
| NLR, median (IQR) | 4.32 (3.42-6.13) | 4.80 (3.15-8.33) | -0.620 | 0.535 |
| PLT, median (IQR), 10^9^/l | 182 (154-218) | 184 (156-251) | -0.814 | 0.416 |
| ALT, median (IQR), U/l | 59.75 (24.68-95.70) | 51.2 (28.3-87) | -0.180 | 0.857 |
| AST, median (IQR), U/l | 48.25 (26.55-86.5) | 54.2 (31.7-81.65) | -0.402 | 0.688 |
| FIB, median (S.D.), g/l | 3.25 (0.80) | 3.38 (0.90) | -0.502 | 0.618 |
| D-Dimer, median (IQR), mg/l | 0.67 (0.45-1.42) | 0.93 (0.49-1.46) | -0.367 | 0.714 |
| IgG, median (IQR), g/l | 13.4 (12.0-17.4) | 12.5 (10.8-15.6) | -1.332 | 0.183 |
| IgA, mean (S.D.), g/l | 3.30 (1.52) | 2.65 (0.99) | 1.545 | 0.140 |
| IgM, median (IQR), g/l | 1.23 (0.85-1.62) | 1.18 (0.90-1.80) | -0.239 | 0.811 |
| LDH, median (IQR), IU/l | 282 (236-336) | 314 (249-404) | -1.199 | 0.231 |
| CK, median (IQR), IU/l | 34 (20-55) | 57 (33-121) | -1.943 | 0.052 |
| C3, median (IQR), g/l | 0.88 (0.70-1.00) | 0.82 (0.73-0.97) | -0.251 | 0.802 |
| C4, mean (S.D.), g/l | 0.24 (0.09) | 0.26 (0.07) | -0.556 | 0.580 |
| CRP, median (IQR), mg/l | 3.1 (2.4-7.5) | 5.2 (2.9-10.6) | -0.917 | 0.359 |
| ESR, median (IQR), mm/h | 40 (23-54) | 33 (16-48) | -0.881 | 0.379 |
| Ferritin, median (IQR), ng/ml | 510.7 (131.2-720.8) | 867.2 (408.3-1206.4) | -1.818 | 0.069 |

P-values were determined using the unpaired t-test, Mann-Whitney U test, χ^2^ test or Fisher’s exact test; **^*^***P* < 0.05, **^**^***P* < 0.01. IB: immunoblotting; WBC: white blood cell; L: lymphocyte; M: monocyte; N: neutrocyte; NLR: neutrophil-lymphocyte ratio; PLT: platelet; ALT: alanine aminotransferase; AST: aspartate aminotransferase; FIB: fibrinogen; LDH: lactic dehydrogenase; CK: creatine kinase; CRP: C-reactive protein; ESR: erythrocyte sedimentation rate.
